# Enhancement in efferocytosis of oxidized low-density lipoprotein-induced apoptotic RAW264.7 cells through Sirt1-mediated autophagy

**DOI:** 10.3892/ijmm.2013.1609

**Published:** 2013-12-27

**Authors:** BAOXIN LIU, BUCHUN ZHANG, RONG GUO, SHUANG LI, YAWEI XU

**Affiliations:** 1 Department of Cardiology, Shanghai Tenth People’s Hospital, Tongji University School of Medicine, Shanghai 200072, P.R. China; 2 Department of Cardiology, The Affiliated Hospital of Xuzhou Medical College, Xuzhou, Jiangsu 221002, P.R. China

**Keywords:** Sirt1, RAW264.7, autophagy, efferocytosis, apoptosis, atherosclerosis

## Abstract

Macrophages play a key role in atherosclerotic plaque formation and rupture. These phagocytic cells are important in the scavenging of modified lipoproteins, unwanted or dead cells and cellular debris through efferocytosis. Sirtuin1 (Sirt1), a member of the conserved sirtuin family and a key regulator in the progression of atherosclerosis exerts protective effects by regulating autophagy, a well-known survival mechanism. Inhibition of autophagy may also result in defective efferocytosis. This study aimed to investigate the effect of Sirt1 on the efferocytosis of oxidized low-density lipoprotein (ox-LDL)-induced apoptotic RAW264.7 cells through upregulation of autophagy. The apoptotic cells were incubated with high and low concentrations of Sirt1 activator resveratrol (RSV) and Sirt1 inhibitor nicotinamide (NAM) as well as autophagy inhibitor 3-methyladenine (3-MA) + low concentration RSV. Apoptosis was determined by flow cytometry (FCM) of annexin-V/propidium iodide (AV/PI) dual staining. Total proteins were extracted and protein levels were detected through western blot analysis. The ox-LDL uptake and efferocytosis of apoptotic RAW264.7 cells were detected by oil red O staining and calculation of the phagocytic index of apoptotic RAW264.7 cells. The expression of Sirt1 and autophagy marker proteins was simultaneously increased with the stimulation of low concentration RSV (all P<0.05) and decreased in low and high NAM groups (all P<0.05), compared with the control group. Efferocytosis was highest in the low concentration RSV group (P<0.001) and relatively lower in the low and high concentration NAM groups (both P<0.05) compared with the control group, which was similar to the change in the expression of Sirt1 and autophagy marker proteins. The results showed that the efferocytosis of apoptotic RAW264.7 cells was significantly improved with the upregulation of Sirt1-mediated autophagy. Therefore, Sirt1 may serve as a novel therapeutic target for the treatment of atherosclerosis.

## Introduction

Atherosclerosis is a chronic immuno-inflammatory disease with high morbidity and atherosclerosis-related cardiovascular diseases are the leading cause of mortality worldwide (1). The destabilization and rupture of atherosclerotic plaques is the main pathological basis of acute cardiovascular disease events without effective treatments. Macrophages play a key role in each stage of atherosclerosis (2). Stimulation of high levels of oxidized low-density lipoprotein (ox-LDL), led to the monocyte-derived macrophages become lipid-laden and are eventually transformed into foam cells. A central feature of atherosclerosis is the accumulation of foam cells in the lesion and, notably macrophage recruitment into plaques is critical for, and increases with, disease progression (3–5). ox-LDL is also a potential inducer of cell apoptosis in atherosclerosis. Previous studies have demonstrated that ox-LDL induced apoptosis in a variety of tissues and cells, including endothelial cells (ECs), vascular smooth muscle cells (VSMCs) and macrophages (6–9). Apoptosis of macrophages and VSMCs in atherosclerotic plaques is thought to lead to increased necrotic core formation, inflammation, plaque rupture and atherothrombosis (10,11). In human atherosclerotic plaques, apoptosis of macrophages is detected during all stages, which occurs more frequently compared with apoptosis of the VSMCs. Accumulating evidence has shown that the phagocytic clearance of apoptotic cells, or ‘efferocytosis’ in macrophages is effective in the early stage of atherosclerosis, whereas efferocytosis in advanced atherosclerosis becomes defective, which is causally linked to the progression of atherosclerosis (12). Therefore, the enhancement in efferocytosis by drugs or other methods in macrophages potentially contributes to the inhibition of atherosclerotic plaques progression and reduction of acute coronary events.

Results of recent studies on macrophage autophagy have shown a novel pathway through which these cells contribute to vascular disease (13–16). Autophagy may be a new target for therapeutic utility in atherosclerosis. Originally described as ‘self-eating’ in the 1960s, autophagy is an evolutionarily conserved controlled cellular catabolic process that mediates the degradation of altered and damaged proteins and organelles. The cellular symbol of autophagy is the formation of characteristic double-membrane vesicles, known as autophagosomes. The origins of this structure remain to be elucidated, although it may be generated from multiple sources including the endoplasmic reticulum (17,18), the outer mitochondrial membrane (17,19), and the plasma membrane (20,21). The autophagosomes are targeted to lysosomes to form single-membraned submicroscopic vesicles termed autolysosomes with degradative capacity. The altered and damaged proteins and organelles were contained in autolysosomes and were eliminated by a series of lysosomal enzymes. Autophagy exerts a protective effect in nutrients generating and maintaining survival (22). Recent evidence suggested that maintenance of basal autophagy in macrophages was useful in the clearance of apoptotic and necrotic cells, which may enhance the efferocytosis of apoptotic macrophages (23–25).

The sirtuins are a family of nicotinamide adenine dinucleotide (NAD)-dependent deacetylases that have been linked to the regulation of life span initially found in yeast cells. Sirtuin1 (Sirt1) is the closest relative of yeast Sir2 in mammalian cells which play important roles in multiple disease-related pathways such as cell cycle regulation, cell apoptosis and migration (26). Resveratrol (3,4′,5-trihydroxy-trans-stilbene, RSV), a polyphenolic phytoalexin, is a potent activator of Sirt1. Nicotinamide (NAM), the precursor for the synthesis of NAD^+^, has been recognized as an inhibitor of Sirt1. Previous results indicated that RSV suppressed atherosclerosis in hypercholesterolemic rabbits and endothelium-specific overexpression of Sirt1 decreased atherosclerosis in apolipoprotein E-deficient (apoE^−/−^) mice. Moreover, Sirt1 modulated neointima formation following vascular injury in mice (27–30). Sirt1 is considered a novel target in the prevention of atherosclerosis by regulating lipid metabolism, promoting endothelial survival and improving endothelial function, repressing vascular smooth muscle cell migration and proliferation and most importantly, inducing cellular autophagy (31–34). Sirt1 was reported to prevent atherosclerosis by potentially regulating the degree of autophagy to match current cellular needs with real-time metabolic status (35).

Focus on VSMCs and ECs has been given in previous investigations on autophagy (23,36,37). Transmission electron microscopy (TEM) of VSMCs in the fibrous cap of experimental or human plaques reveals features of autophagy such as the formation of myelin figures (36). Moreover, experimental exposure of endothelial cells directly to ox-LDL strongly enhanced autophagy compared with exposure to LDL only, indicating that autophagy may contribute to the degradation of ox-LDL (37,38). Autophagy has also been reported to regulate intracellular lipid metabolism to avoid the formation of foam cells (39,40). As recently reviewed (41,42), lipophagy, a special type of autophagy, contributes to cholesterol egress from lipid-laden cells to high-density lipoprotein (HDL) via lysosomal lipases. Autophagy can play a role in the hydrolysis of stored cholesterol droplets in macrophages, thus facilitating cholesterol efflux (43). The function of macrophage autophagy in atherosclerosis has been complicated by the strong phagocytic activity of these cells. Currently, few studies have focused on the enhancement in efferocytosis of apoptotic macrophages through Sirt1-mediated autophagy. However, autophagy is a ‘double-edged sword’. The general consensus is that basal autophagy can protect plaque cells against oxidative stress by degrading damaged intracellular material and promoting cell survival, and excessive stimulation of autophagy may cause autophagic cell death, leading to reduced synthesis of collagen, thinning of the fibrous cap, plaque destabilization, lesional thrombosis, and acute clinical events (44,23,24). Thus the aim of this study was to determine whether there was a connection between enhancement in efferocytosis of apoptotic macrophages and autophagy mediated by Sirt1.

## Materials and methods

### Reagents and antibodies

A Cell Counting Kit-8 (CCK-8) was purchased from Dojindo Laboratories (Kumamoto, Japan). ox-LDL was purchased from AbD Serotec (Kidlington, Oxford, UK). Oil red O staining, 5(6)-carboxyfluorescein diacetate N-succinimidyl ester (CFSE) staining, Sirt1-activator RSV, Sirt1-inhibitor NAM and autophagy-inhibitor 3-methyladenine (3-MA) were purchased from Sigma-Aldrich Co. (St. Louis, MO, USA). The anti-Sirt1 antibody was obtained from Cell Signaling (Beverly, MA, USA). The anti-light chain (LC) 3, anti-Atg5 and anti-Atg7 antibodies were obtained from Epitomics (Burlingame, CA, USA). Horseradish peroxidase-linked anti-rabbit, anti-mouse secondary antibodies and RIPA buffer were obtained from Santa Cruz Biotechnology, Inc. (Santa Cruz, CA, USA).

### Cell culture and experimental design

A mouse macrophage-like RAW264.7 cell line was purchased from the Cell Bank of the Shanghai Institutes for Biological Sciences, Chinese Academy of Sciences. The cells were cultured in Dulbecco’s modified Eagle’s medium (DMEM, Gibco, NY, USA), which contained 5 mM glucose, and were supplemented with 10% fetal bovine serum (FBS) (Gibco) and 1% penicillin/streptomycin at 37°C with 5% CO_2_ in a humidified atmosphere. The cells were passaged every 2–3 days and then placed into 6-well plates with slides at a density of 4×10^5^ cells/cm^2^ and made apoptotic by incubation with ox-LDL. The optimal concentration and time point of ox-LDL were analyzed using western blotting of Sirt1 and the autophagy marker proteins. The apoptotic cells were randomly divided into the following groups: i) control group, only apoptotic cells, ii) low concentration RSV group: apoptotic cells incubated with RSV of low concentration, iii) high concentration RSV group: apoptotic cells incubated with RSV of high concentration, iv) low concentration NAM group: apoptotic cells incubated with NAM of low concentration, v) high concentration NAM group: apoptotic cells incubated with NAM of high concentration, vi) 3-MA + low concentration RSV group: the apoptotic cells were pretreated with 10 mM 3-MA for 2 h, and then incubated with low concentration RSV. The low and high concentration of RSV and NAM were assayed from the cell proliferation of RAW264.7 incubation with RSV and NAM for 24 h.

### Protein extraction and western blot analysis

Total proteins were obtained by rinsing treated cells with ice-cold phosphate-buffered saline (PBS), and lysing in lysis buffer (10 mM Tris pH 7.4, 20 mM NaCl, 5 mM MgCl_2_, 0.5% NP-40 and 0.1 mM PMSF). The extracts were then centrifuged at 12,000 × g for 10 min at 4°C, and the clear supernatants containing total protein were collected. The protein concentration was measured with the Bio-Rad protein assay (Hercules, CA, USA), an equal amount of protein was separated on SDS-polyacrylamide gel electrophoresis and transferred to nitrocellulose membranes. After blocking with 5% non-fat milk, the membranes were incubated overnight with the previously mentioned first antibodies at 4°C. The membranes were then incubated with the appropriate secondary antibodies, and the bands were detected by enhanced chemiluminescence. The band density value was quantified using the ImageJ image processing program. Since the extent of conversion of LC3-I to LC3-II is correlated with the level of autophagy, LC3-I and LC3-II were detected by western blot analysis, and the conversion of LC3 was demonstrated by LC3-II/LC3-I ratio. The LC3-II/LC3-I ratio was calculated as follows: LC3-II/LC3-I ratio = the band density of LC3-II/the band density of LC3-I. We also detected the expression of two separate autophagy proteins, Atg5 and Atg7, which were ultimately required for the formation of the autophagosome and the subsequent induction of autophagy (45–47).

### Cell Counting Kit-8 assay

Cell proliferation was assayed using a CCK-8 assay. Cell suspension (100 μl) of RAW264.7 cells seeded in 96-well plates at a density of 1×10^5^ cells/well was performed. RAW264.7 cells were then treated with RSV or NAM. The final concentrations of RSV were 50, 25, 12.5, 5 and 0 μM, respectively. The final concentrations of NAM were 80, 40, 20, 10, 5 and 0 mM, respectively. Each group was prepared with five parallel wells and incubated at 37°C, 5% CO_2_, for 24 h. At the end of the culture period, CCK-8 was added to each well according to the manufacturer’s instructions at a final concentration of 0.5 mg/ml for 3 h. The absorbance was measured with an enzyme calibrator at 450 and 650 nm and optical density (OD) values were measured. Experiments were repeated three times.

### Oil red O staining

The RAW264.7 cells, measured for lipid accumulation through staining of ox-LDL with oil red O, were placed in 6-well plates with slides at a density of 4×10^5^ cells/cm^2^ and followed with the aforementioned treatments. At the end of the treatment period, the cells were rinsed with PBS and fixed with 10% formalin for 5 min at room temperature. The cells were then rinsed again with 60% isopropanol and incubated with fresh-filtered oil red O solution (60% saturated oil red O/40% deionized water) for 20 min. For analysis, the slides were washed in isopropanol for 10 min, rinsed in distilled water, counterstained with hematoxylin and mounted in glycerol/gelatin solution. Images of cells were captured using a fluorescence microscope.

### Analysis of apoptosis by flow cytometry (FCM) of Annexin-V/propidium iodide (AV/PI) dual staining

The RAW264.7 cells were incubated with ox-LDL of designed concentrations and were then processed with an AV-FITC kit (Keygene, KGA108) according to the manufacturer’s instructions. The samples were analyzed by FACScan flow cytometer (Becton-Dickinson, Franklin Lakes, NJ, USA) in order to quantify the apoptotic rate. Different subpopulations were distinguishable: Q1, Annexin V-negative but PI-positive, i.e., necrotic cells; Q2, Annexin V/PI-double positive, i.e., late apoptotic cells; Q3, Annexin V/PI-double negative, i.e., live cells; Q4, Annexin V-positive but PI-negative, i.e., early apoptotic cells. The apoptotic rate was determined as the percentage of Q2 + Q4.

### Detection of autophagosomes by TEM analysis

The treated cells were rinsed with ice-cold PBS and centrifuged at 1,000 × g for 5 min at room temperature, after which the clear supernatants were removed. Cell pellets were fixed with 2.5% glutaraldehyde in 0.1 M cacodylate buffer, pH 7.4 for at least 30 min at 4°C. After fixation, the specimens were thoroughly washed in 0.1 M cacodylate buffer and then post-fixed with 1% osmium tetroxide in the same buffer for 1 h at room temperature. The specimens were dehydrated in a graded series of ethanol, and embedded in Epon, then 0.1 μm thin sections were stained with uranyl-acetate/lead citrate and viewed in a Hitachi H-300 TEM.

### Measurement of the efferocytosis of apoptotic RAW264.7 cells

The measurement of the efferocytosis of RAW264.7 cells was performed as described by Li *et al,* Yancey *et al* and Jehle *et al* (48–50). The RAW264.7 cells were made apoptotic by incubation with ox-LDL followed by treatments in the aforementioned 6 groups. After vigorous washing with PBS, the cells were fixed in 4% paraformaldehyde and counterstained with PI. The cells in 6 groups were incubated for 2 h with fresh RAW264.7 cells which were labeled with CFSE cell tracer. The efferocytosis of apoptotic RAW264.7 cells was visualized using fluorescence microscopy. PI red-labeled apoptotic RAW264.7 cells merged into CFSE cell tracer green-labeled fresh RAW264.7 cells, which was considered as phagocytosis of the apoptotic cells by fresh RAW264.7 cells. The phagocytic index was used to evaluate the efferocytosis of apoptotic RAW264.7 cells. The phagocytic index was calculated using the formula: Phagocytic index = (number of phagocytized RAW264.7 cells/number of total cells) × 100. Experiments were repeated five times and the analysis was performed in a blinded fashion by two independent observers.

### Statistical analysis

Data are expressed as mean ± SD. Statistical analysis of data was performed by applying the Student’s t-test to determine the significance between two groups. Statistical significance of pairwise differences among three or more groups were determined using one-way analysis of variance (ANOVA) followed by post-hoc test. P<0.05 was considered statistically significant. Analysis was performed using SPSS for Windows (SPSS Inc., Version 16.0, Chicago, IL, USA).

## Results

### Expression of Sirt1 and autophagy marker proteins was elevated at optimal concentrations and time point of ox-LDL

**Figure 1:**
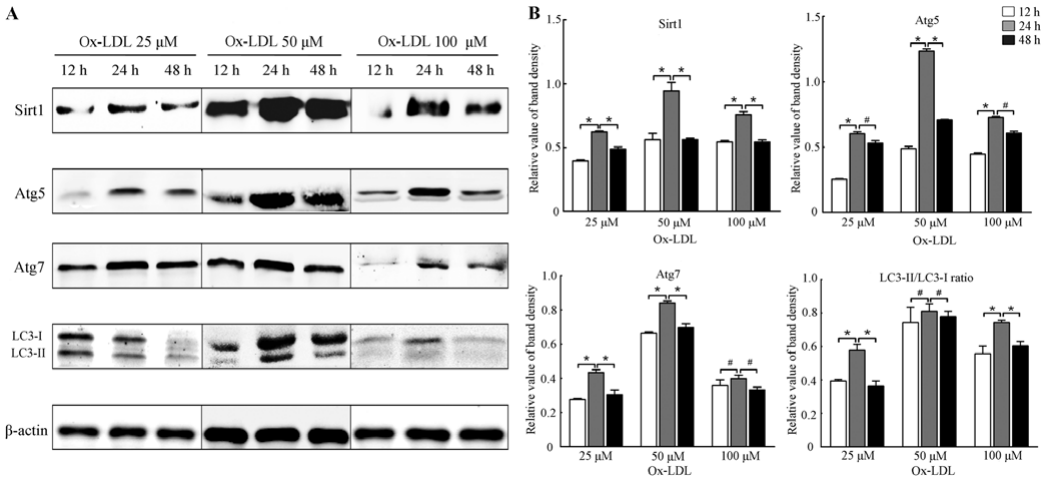
Expression of Sirtuin1 (Sirt1) and autophagy marker proteins in RAW264.7 cells treated with ox-LDL at different concentrations and time points. (A) Representative results of assays of Sirt1, Atg5, Atg7, LC3-I and LC3-II and β-actin abundances in RAW264.7 cells with ox-LDL of designated concentrations (25, 50 and 100 μM) and time points (12, 24 and 48 h) using western blot analysis. (B) Protein expression levels of Sirt1 were analyzed by western blotting by using polyclonal antibodies to Sirt1, Atg5, Atg7, LC3-I and LC3-II, respectively, in order to quantify the expression in RAW264.7 cells. LC3-II/LC3-I ratio was calculated. β-actin was used as an equal loading control. The band value was quantified by densitometric analysis. The expression of all the proteins was increased most at 24 h compared with 12 or 48 h (all P<0.05). Experiments were repeated at least three times. Data are expressed as the mean ± SD in the corresponding bar graph and statistical significance was determined by the Student’s t-test. Columns, mean; error bars, ±SD; ^#^P<0.05, ^*^P<0.01.

**Figure 2:**
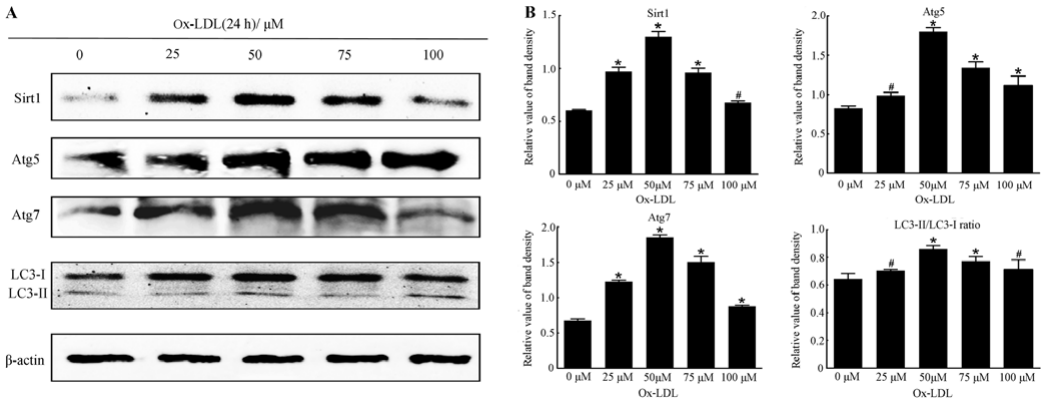
The optimal concentration of ox-LDL for the expression of Sirtuin1 (Sirt1) and autophagy marker proteins in RAW264.7 cells after incubation for 24 h. (A) Representative western blot analysis results of Sirt1, Atg5, Atg7, LC3-I and LC3-II and β-actin abundances in RAW264.7 cells following stimulation with different concentrations of ox-LDL (0, 25, 50, 75 and 100 μM) for 24 h. (B) Protein levels of Sirt1, Atg5, Atg7, LC3-I and LC3-II were analyzed by western blotting in RAW264.7 cells, respectively. The LC3-II/LC3-I ratio was calculated. β-actin was used as an equal loading control. The band value was quantified by densitometric analysis. The expression of all the proteins was increased after incubation with ox-LDL at the designated concentrations compared with 0 μM ox-LDL (all P<0.05). The optimal concentration of ox-LDL was 75 μM compared with the other groups. Experiments were repeated at least three times. Data are expressed as the mean ± SD in the corresponding bar graph and statistical significance was determined by the Student’s t-test. Columns, mean; error bars, ±SD; ^#^P<0.05 vs. 0 μM group, ^*^P<0.01 vs. 0 μM group.

The effects of ox-LDL (25, 50 and 100 μM) on the expression of Sirt1 and autophagy marker proteins at different time points (12, 24 and 48 h) were examined. Our results showed that ox-LDL of appropriate concentration elevated the levels of Sirt1 and autophagy marker proteins such as Atg5, Atg7 and LC3-II/LC3-I at optimal time points. Results of the western blot analysis shown in Figs. 1 and 2 revealed that the expression of Sirt1 and autophagy marker proteins was increased at 24 h (all P<0.05 vs. 12 h), and then decreased at 48 h (all P<0.05 vs. 24 h). The expression of Sirt1 and autophagy marker proteins was significantly higher at 50 μM ox-LDL (all P<0.05 vs. 0 μM), but was reduced when the cells were treated with 75 and 100 μM ox-LDL (all P<0.05 vs. 0 μM). Thus, cells treated with 50 μM ox-LDL for 24 h may be considered optimal for the expression of Sirt1 and autophagy marker proteins. Moreover, the results suggested that autophagy was induced concomitantly with the induction of expression of Sirt1 by a moderate stimulus of ox-LDL, suggesting that Sirt1 is involved in autophagy under treatment of ox-LDL to some extent.

### Ox-LDL treatment simultaneously induces autophagy and apoptosis in RAW264.7 cells

**Figure 3:**
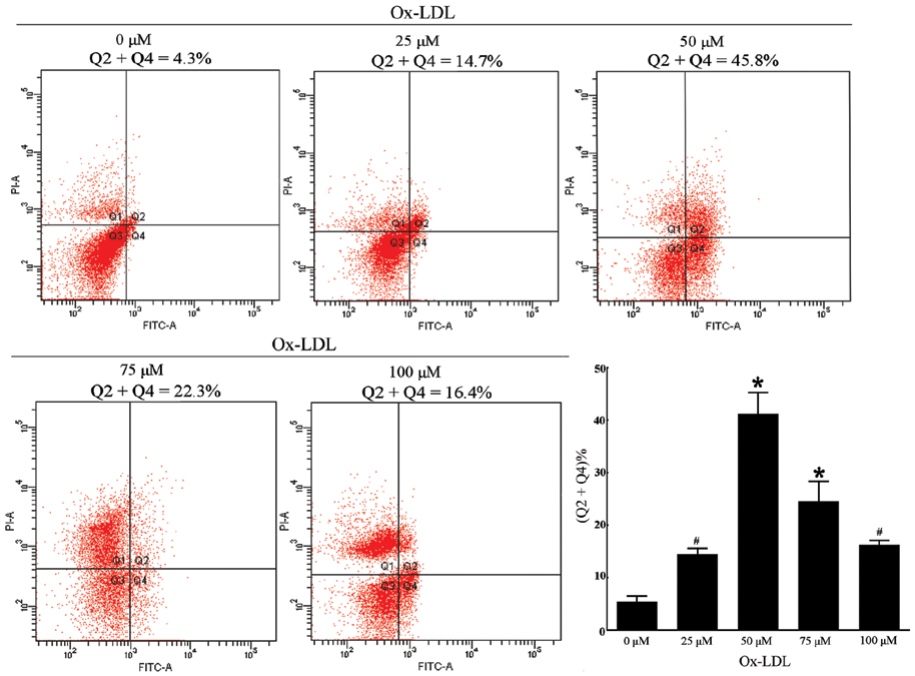
Quantitative analysis of apoptosis by flow cytometry (FCM) of Annexin-V/propidium iodide (AV/PI) dual staining in ox-LDL-treated RAW264.7 cells. The cells were treated with different concentrations of ox-LDL (0, 25, 50, 75 and 100 μM) for 2 h. The results showed that the apoptotic rates were all increased after treatment with ox-LDL at the designated concentrations (all P<0.05 vs.), but increased most in the 50 μM group (P<0.001 vs. 0 μM group). Experiments were repeated three times. Data are expressed as the mean ± SD in the corresponding bar graph and statistical significance was determined by Student’s t-test. Columns, mean; error bars, ±SD; ^#^P<0.05 vs. 0 μM group, ^*^P<0.01 vs. 0 μM group.

We investigated cell apoptosis when the cells were incubated with different concentrations of ox-LDL for 24 h. An appropriate concentration of ox-LDL for further measurement of efferocytosis was required according to the apoptotic rate. Additionally, the quantitative analysis of apoptosis by FCM of AV/PI dual staining at designated concentrations showed that the apoptotic rate increased at 25 μM (14.52±1.08% vs. 0 μM, 5.43±1.04%) and 50 μM (41.23±4.02%) in a dose-dependent manner and markedly accelerated when treated with 50 μM ox-LDL (Fig. 3). The apoptotic rate decreased when the cells were incubated with 75 μM (24.53±3.82%) and 100 μM (16.30±0.79%) ox-LDL. By comparing the apoptotic rate among these concentrations, the data suggested that the apoptotic rate was appropriate when the cells were treated with 50 μM ox-LDL (P<0.001 vs. 0 μM). Furthermore, the previous results showed that, cells treated with 50 μM ox-LDL for 24 h would be optimal for the expression of autophagy marker proteins, which is similar to the condition of cell apoptosis. The evidence suggested that autophagy and apoptosis of RAW264.7 cells were triggered by incubation with 50 μM ox-LDL for 24 h.

### Different doses of RSV and NAM exert dual effects on cell proliferation and the expression of Sirt1 in RAW264.7 cells

**Figure 4:**
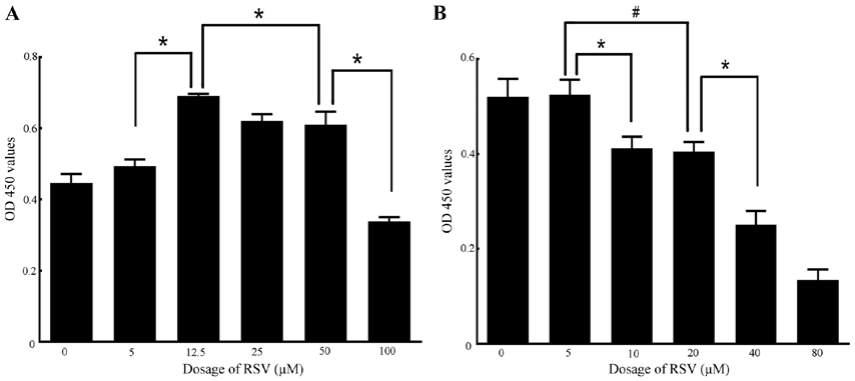
Determination of high and low concentrations of resveratrol (RSV) or nicotinamide (NAM) by analysis of cell proliferation rates using the Cell Counting Kit-8 (CCK-8) assay. (A) Results of CCK-8 assay on RAW264.7 cells incubated with different doses of RSV (0, 5, 12.5, 25, 50 and 100 μM). No significant differences were observed in comparisons between 0 and 5 μM, and 25 and 50 μM groups, respectively. A significant difference was observed between the 5 and 12.5 μM, 12.5 and 50 μM group, and 50 and 100 μM groups, respectively (all P<0.01). (B) Results of CCK-8 assay on RAW264.7 cells incubated with different doses of NAM (0, 5, 10, 20, 40 and 80 mM). No significant differences were shown in comparisons between the 0 and 5 mM, and 10 and 20 mM groups, respectively. A significant difference was observed between the 5 and 10 mM, 5 and 20 mM, and 20 and 40 mM groups, respectively (all P<0.05). Experiments were repeated three times. Data are expressed as the mean ± SD in the corresponding bar graph and statistical significance was determined by the Student’s t-test. Columns, mean; error bars, ±SD; ^#^P<0.05, ^*^P<0.01.

**Figure 5:**
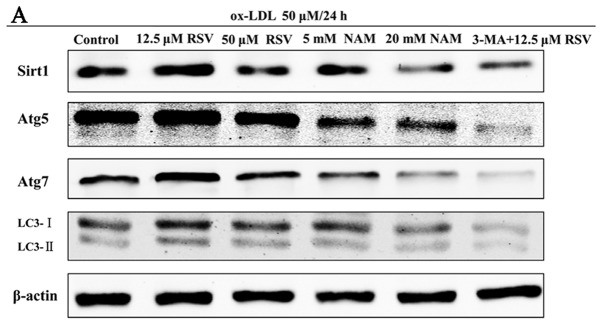

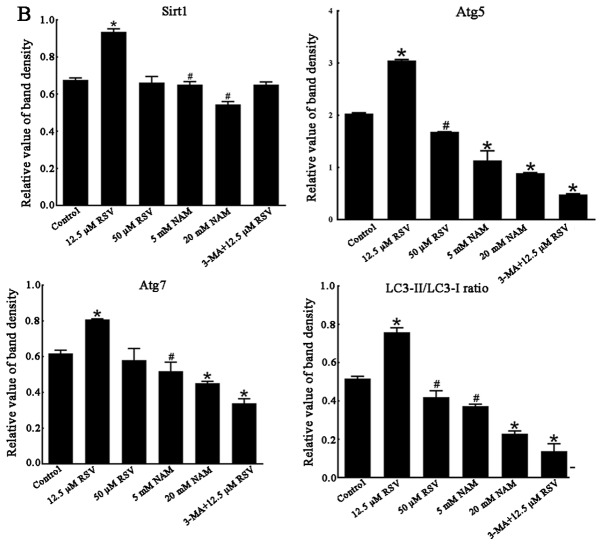
Expression of Sirtuin1 (Sirt1) and autophagy marker proteins in ox-LDL-induced apoptotic RAW264.7 cells of different treatments. The apoptotic cells were randomly divided into 6 groups: i) control, ii) 12.5 μM resveratrol (RSV), iii) 50 μM RSV, iv) 5 mM nicotinamide (NAM), v) 20 mM NAM, vi) 3-methyladenine (3-MA) + 12.5 μM RSV. (A) Representative results of assays of Sirt1, Atg5, Atg7, LC3-I, LC3-II and β-actin abundances of 6 groups using western blot analysis. (B) The levels of Sirt1, Atg5, Atg7, LC3-I and LC3-II protein expression were analyzed by western blot analysis by using polyclonal antibodies to Sirt1, Atg5, Atg7, LC3-I and LC3-II to quantify the expression in these groups. LC3-II/LC3-I ratio was calculated. Sirt1 expression was significantly elevated in 12.5 μM RSV group, compared with control group (P=0.002). No statistical significance was observed between the 3-MA + 12.5 μM RSV and control groups following inhibition of autophagy. The levels of all autophagy marker proteins were significantly increased in 12.5 μM RSV group simultaneously, compared with the control group (all P<0.05). β-actin was used as an equal loading control. The band value was quantified by densitometric analysis. Experiments were repeated at least three times. Data are expressed as the mean ± SD in the corresponding bar graph and statistical significance was determined by the Student’s t-test. Columns, mean; error bars, ±SD; ^#^P<0.05 vs. control group, ^*^P<0.01 vs. control group.

Low concentration RSV exerted a protective effect on cells. However, high concentration RSV may induce cell necrosis and apoptosis of RAW264.7 cells (51). NAM of different concentrations was also able to promote or inhibit cell proliferation (52,53). The dual effects of RSV and NAM on cell proliferation and the expression of Sirt1 in RAW264.7 cells were examined. The appropriate high and low concentrations of RSV or NAM for cell proliferation, respectively, were identified. The results of CCK-8 assay indicated that the cell proliferation rates increased significantly when incubated with 12.5 μM RSV (0.69±0.01 OD), compared with that of the 0 μM group (0.45±0.03 OD), 5 μM group (0.49±0.02 OD), 25 μM group (0.62±0.02 OD), 50 μM group (0.61±0.04 OD) and 100 μM group (0.34±0.01 OD). The proliferation rates decreased significantly when the cells incubated with 100 μM RSV (0.34±0.01 OD vs. 50 μM: 0.61±0.04 OD) (Fig. 4A). Similarly, the cell proliferation rates remained appropriate when incubated with 5 mM NAM (0.52±0.03 OD) compared with 0 mM NAM (0.52±0.04 OD). The proliferation rates decreased in the 10 mM NAM group (0.41±0.02 OD), 20 mM NAM group (0.40±0.02 OD), and most significantly, the 40 mM NAM (0.25±0.03 OD) and 80 mM NAM groups (0.14±0.02 OD) (Fig. 4B). These results suggested that the cell status was extremely poor when incubated with 100 μM RSV, 40 and 80 mM NAM. Thus, the high and low concentrations of RSV were 50 and 12.5 μM, while the corresponding high and low concentrations of NAM were 20 and 5 mM. Furthermore, after the RAW264.7 cells were made apoptotic, the results of western blotting revealed that Sirt1 expression was increased when cells were incubated with 12.5 μM RSV and 5 mM NAM compared with that of 50 μM RSV and 20 mM NAM, respectively (both P<0.05 vs. control) (Fig. 5A and B).

### Sirt1 possibly contributes to autophagy in apoptotic RAW264.7 cells following treatment of ox-LDL

**Figure 6:**
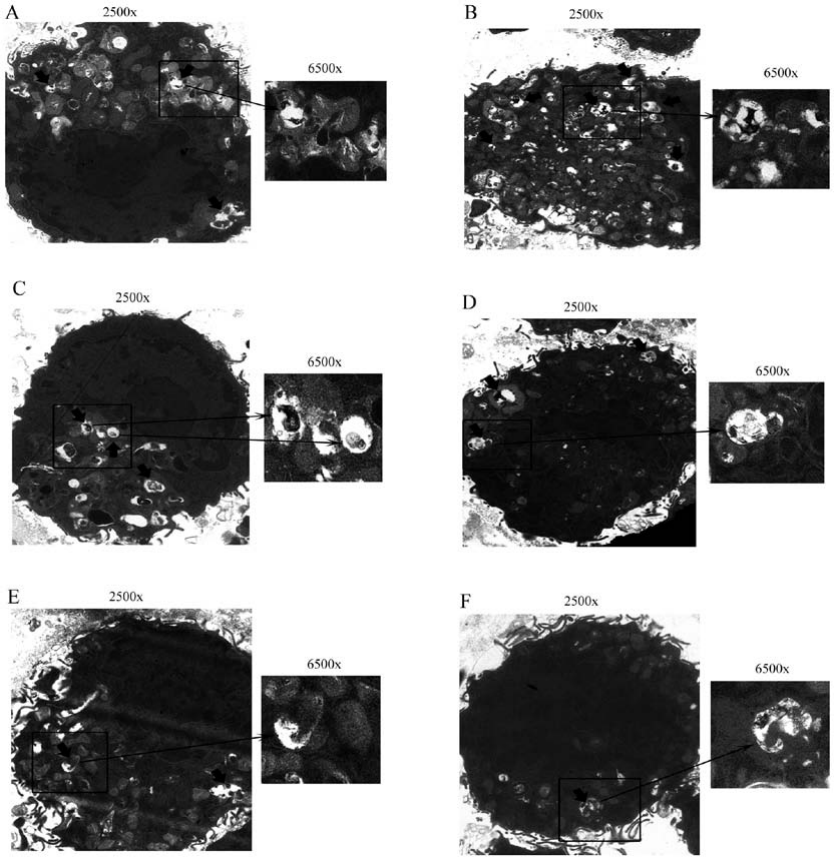
Representative results of the formation and structure of autophagosomes in ox-LDL-induced apoptotic RAW264.7 cells of different treatments. The autophagosomes were detected by TEM. (A) Control group, (B) 12.5 μM resveratrol (RSV) group, (C) 50 μM RSV group, (D) 5 mM nicotinamide (NAM) group, (E) 20 mM NAM group and (F) 3-methyladenine (3-MA) + 12.5 μM RSV group. The formation of autophagosomes was detected in each group. However, there were significantly more autophagosomes in the 12.5 μM RSV group. Inhibition of autophagy resulted in a significant reduction of autophagosomes in the 3-MA + 12.5 μM RSV group. Experiments were repeated five times. Black arrow, autophagosome.

To define the potential role of Sirt1 in autophagy, we examined the expression of Sirt1 and autophagy marker proteins in apoptotic RAW264.7 cells following the treatment of 50 μM ox-LDL for 24 h, using high and low concentration RSV or NAM. We also investigated the expression of these proteins when the cells were incubated with 3-MA, the chemical inhibitor of autophagy. Treatment with 12.5 μM RSV significantly increased the expression of autophagy marker proteins such as Atg5, Atg7 and LC3-II/LC3-I, which was accompanied by the activation of Sirt1 (all P<0.05 vs. control group). The expression of Sirt1 and autophagy marker proteins in the 5 and 20 mM NAM groups were simultaneously decreased as compared to the control group (all P<0.05) (Fig. 5). Moreover, the expression of Sirt1 in the 3-MA + 12.5 μM RSV group showed no significant difference compared with the control group (P=0.07) subsequent to inhibition of autophagy (Fig. 5A and B). We also detected the autophagosomes of these groups via TEM analysis. Significantly more autophagosomes were identified in the 12.5 μM RSV group compared with the other groups, while there was hardly any formation of autophagosomes in the 3-MA + 12.5 μM RSV group (Fig. 6). These results showed that Sirt1 was able to regulate the expression of autophagy marker proteins in RAW264.7 cells.

### Upregulation of autophagy enhanced efferocytosis of apoptotic RAW264.7 cells

**Figure 7:**
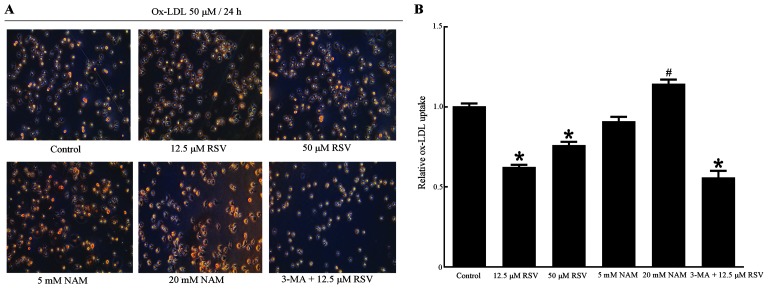
The ox-LDL uptake of apoptotic RAW264.7 cells of different treatments. (A) Representative results of oil red O staining in ox-LDL-induced apoptotic RAW264.7 cells of different treatments. Images were obtained from a fluorescence microscope. Red fluorescence represents the accumulation of ox-LDL in cells. (B) Analysis of ox-LDL uptake in apoptotic RAW264.7 cells of different treatments. Results were shown as fold changes in the proportions of the oil red O-stained positive area compared with the control. The ox-LDL uptake in the 12.5 and 50 μM resveratrol (RSV) group was decreased (P<0.001, P=0.008 vs. control group respectively), and increased in the 20 mM group (P=0.03 vs. control group). The ox-LDL uptake was also decreased in the 3-methyladenine (3-MA) + 12.5 μM RSV group compared with the control group (P<0.001), which was possibly caused by elevation in the level of Sirtuin1 (Sirt1) although the autophagy was inhibited. Experiments were repeated five times. Data are expressed as the mean ± SD in the corresponding bar graph and statistical significance was determined by the Student’s t-test. Columns, mean; error bars, ±SD; ^#^P<0.05 vs. control group, ^*^P<0.01 vs. control group.

**Figure 8:**
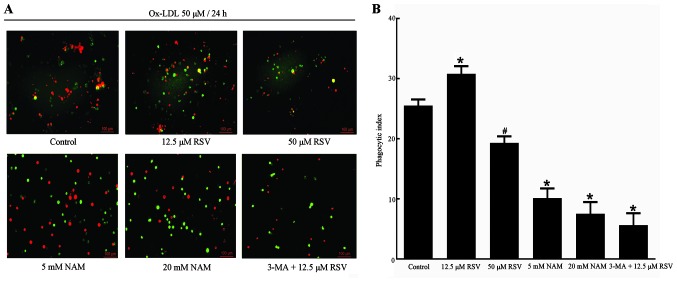
Measurement of the efferocytosis of ox-LDL-induced apoptotic RAW264.7 cells of different treatments. (A) Representative results of the efferocytosis of ox-LDL-induced apoptotic cells of different treatments. Images were obtained from a fluorescence microscope. Red fluorescence represents ox-LDL-induced apoptotic RAW264.7 cells using a PI staining. Green CFSE staining fluorescence represents fresh RAW264.7 cells. (B) Analysis of phagocytic index in apoptotic RAW264.7 cells of different treatments. The phagocytic index 12.5 μM resveratrol (RSV) group was markedly increased compared with the control group (P<0.001), indicating that efferocytosis of apoptotic RAW264.7 cells was enhanced by Sirt1-mediated autophagy. The efferocytosis of apoptotic RAW264.7 cells was decreased in the 5 mM nicotinamide (NAM), 20 mM NAM and 3-MA + 12.5 μM RSV groups (all P<0.001 vs. control group). Data are expressed as the mean ± SD in the corresponding bar graph and statistical significance was determined by the Student’s t-test. Experiments were repeated five times. Columns, mean; error bars, ±SD; ^#^P<0.05 vs. control group, ^*^P<0.01 vs. control group.

The relationship between Sir1-mediated autophagy and efferocytosis in apoptotic RAW264.7 cells was investigated. ox-LDL uptake was detected, which may be useful in the elevation of the phagocytosis of RAW264.7 cells regulated by Sirt1 and autophagy. As determined by oil red O staining (Fig. 7), ox-LDL uptake in the 12.5 and 50 μM RSV groups were decreased compared with the control group (P<0.001, P=0.008 respectively), which was accompanied by the upregulation of autophagy. By contrast, ox-LDL uptake in the 20 mM group NAM showed a significant increase (P=0.03 vs. control group), whereas a decrease was identified in the 3-MA + 12.5 μM RSV group following the inhibition of autophagy (P<0.001 vs. control group). This finding may be attributed to the expression of Sirt1 since Sirt1 has been reported to decrease ox-LDL uptake and prevent macrophage foam cell formation through suppression of the expression of the scavenger receptor Lox-1 in macrophages (54). Another possible reason is the poor status of the cells in this group, since there were significant fewer cells compared with the other groups, as noted in the images obtained via fluorescence microscopy (Figs. 7A and 8A). The results from the measurement of the efferocytosis demonstrated that 12.5 μM RSV caused a marked increase in the efferocytosis of apoptotic RAW264.7 cells compared with the control group (P<0.001) (Fig. 8), suggesting that upregulation of autophagy contributes to the phagocytic clearance of apoptotic cells. Similarly, the efferocytosis of apoptotic RAW264.7 cells was decreased in the 5 and 20 mM NAM groups (P=0.001, P<0.001 respectively vs. the control group). It is also likely that inhibition of autophagy by 3-MA contributes to defective efferocytosis, although Sirt1 was expressed. All our results showed that upregulation of autophagy was capable of enhancing efferocytosis of apoptotic RAW264.7 cells. Since the expression of Sirt1 was able to regulate autophagy and the improvement of efferocytosis was accompanied by an increase in the expression of Sirt1, the enhancement in efferocytosis of apoptotic RAW264.7 cells could be Sirt1-mediated. However, only the expression of Sirt1 did not induce enhancement in efferocytosis following inhibition of autophagy.

## Discussion

The main aim of this study was to determine whether the upregulation of autophagy mediated by Sirt1 could enhance efferocytosis of apoptotic macrophages induced by ox-LDL. The expression of Sirt1 and autophagy marker proteins was investigated, using Sirt1 activator RSV and inhibitor NAM. The findings in our study suggest that with the increase in the expression of Sirt1 activated by an appropriate dose of RSV, the expression of autophagy marker proteins was also increased. Furthermore, the efferocytosis of apoptotic RAW264.7 cells was also improved simultaneously with this increase.

Previous studies (13–16) have shown that macrophage autophagy in atherosclerosis becomes dysfunctional in atherosclerosis and its deficiency promotes vascular inflammation, oxidative stress, and plaque necrosis, suggesting a mechanism-based strategy to therapeutically suppress atherosclerosis progression. It is difficult to determine whether the vacuoles in their cytoplasm result from autophagocytosis or heterophagocytosis through conventional electron microscopy analysis. In addition, LC3 is poorly expressed in macrophages and overexpression of other lysosomal marker proteins may give rise to false-positive signals in immunoelectron microscopy (23,24). Notably, pharmacological modulation of macrophage autophagy has been shown to affect vascular inflammation. Stent-based delivery of everolimus (a well-known autophagy inducer) in atherosclerotic plaques of high-fat diet-fed rabbits leads to a marked reduction of macrophages via autophagic cell death without altering the VSMC plaque content (13). Two recent studies have provided new dimensions to the understanding of the role of macrophage autophagy in regulating atherosclerosis. Razani and colleagues (15) demonstrated that initially autophagy is functional and becomes severely compromised with disease progression. This deficiency of macrophage autophagy may induce inflammosome hyperactivation through lysosomal leakage, generation of reactive oxygen species (ROS), and impaired mitophagy, thus increased vascular inflammation and plaque formation in apoE^−/−^ mice. Findings of another study provided evidence that inhibition of macrophage autophagy enhanced apoptosis and riphosphopyridine nucleotide (NADPH) oxidase-mediated oxidative stress, rendering the apoptotic cells less recognizable to efferocytosis in low-density lipoprotein receptor (LDLr)^−/−^ mice (16). The defective efferocytosis could promote plaque necrosis in advanced atherosclerosis. These data indicated a protective role played by macrophage autophagy in the two most widely used mouse models of atherosclerosis.

Sirt1 has been recently regarded as a new factor in the regulation of autophagy. The decrease in Sirt1 protein expression may lead to inflammation through dysregulation of autophagy and increased levels of acetylated nuclear factor-κB (NF-κB) (55,56). Sirt1 regulated autophagy by promoting the formation of autophagosome in a cellular model of oxidative stress (57). Moreover, RSV exerted protective effects on cells through the activation of the adenosine 5′-monophosphate-activated protein kinase (AMPK)-SIRT1-autophagy pathway. Inhibition of Sirt1 contributes to the dysregulation of other nutrient-sensing pathways including mammalian target of rapamycin (mTOR) and AMPK, thereby leading to the impairment of autophagy in human macrophage, which may induce inflammation through NF-κB activation and the accumulation of autophagy marker proteins (58,59). However, few studies have explored the relationship between efferocytosis of apoptotic macrophage cells and autophagy mediated by Sirt1. Our results mainly provide preliminary evidence that Sirt1 potentially affects the efferocytosis of apoptotic macrophages via the activation of autophagy, which is useful in understanding the molecular mechanism and pathogenesis of atherosclerosis. Our findings may have implications regarding the influence of defective autophagy on cell survival and the expression of Sirt1 in atherosclerosis, since the cell status was poor and the level of Sirt1 was not elevated when the cells were incubated with 3-MA + 12.5 μM RSV.

Limitations to our study should be noted. The study was performed using *in vivo* experimental systems. Additionally, the relationship between efferocytosis of apoptotic macrophages and Sirt1 should be confirmed in the atherosclerotic plaques through *in vitro* experiments. We only detected the efferocytosis of apoptotic macrophages enhanced by Sirt1-mediated moderate autophagy. Thus, the effect of Sirt1-mediated excessive autophagy on efferocytosis of apoptotic macrophages should also be determined. Studies are also required to elucidate the signaling pathways underlying this mechanism.

In conclusion, results of the present study showed that autophagy was upregulated by an appropriate dose of Sirt1 activator RSV, and that the efferocytosis of apoptotic RAW264.7 was significantly improved when incubated with the appropriate dose of RSV compared with Sirt1 inhibitor NAM and autophagy inhibitor 3-MA. This enhancement in efferocytosis may be associated with Sirt1-mediated autophagy.
